# The Role of Nurses and Community Health Workers in Confronting Neglected Tropical Diseases in Sub-Saharan Africa: A Systematic Review

**DOI:** 10.1371/journal.pntd.0004914

**Published:** 2016-09-15

**Authors:** Andrew G. Corley, Clifton P. Thornton, Nancy E. Glass

**Affiliations:** 1 Department of Community-Public Health, Johns Hopkins University School of Nursing, Baltimore, Maryland, United States of America; 2 Johns Hopkins University School of Medicine, Baltimore, Maryland, United States of America; Swiss Tropical and Public Health Institute, SWITZERLAND

## Abstract

**Introduction:**

Neglected tropical diseases produce an enormous burden on many of the poorest and most disenfranchised populations in sub-Saharan Africa. Similar to other developing areas throughout the world, this region’s dearth of skilled health providers renders Western-style primary care efforts to address such diseases unrealistic. Consequently, many countries rely on their corps of nurses and community health workers to engage with underserved and hard-to-reach populations in order provide interventions against these maladies. This article attempts to cull together recent literature on the impact that nurses and community health workers have had on neglected tropical diseases.

**Methods:**

A review of the literature was conducted to assess the role nurses and community health workers play in the primary, secondary, and tertiary prevention of neglected tropical diseases in sub-Saharan Africa. Articles published between January 2005 and December 2015 were reviewed in order to capture the full scope of nurses’ and community health workers’ responsibilities for neglected tropical disease control within their respective countries’ health systems.

**Results:**

A total of 59 articles were identified that fit all inclusion criteria.

**Conclusions:**

Successful disease control requires deep and meaningful engagement with local communities. Expanding the role of nurses and community health workers will be required if sub-Saharan African countries are to meet neglected tropical disease treatment goals and eliminate the possibility future disease transmission. Horizontal or multidisease control programs can create complimentary interactions between their different control activities as well as reduce costs through improved program efficiencies—benefits that vertical programs are not able to attain.

## Introduction

Neglected tropical diseases (NTDs) are the most common pathogens suffered by 500 million of the poorest people living in sub-Saharan Africa (SSA). This group of little-known diseases with oftentimes difficult to pronounce names takes an enormous toll on public health (8.6–21.2 million disability-adjusted life years [DALYs]) and is estimated to create a disease burden possibly as great as half that of malaria (40.9 million DALYs) and twice that of tuberculosis (9.3 million DALYs) [1]. The great tragedy of these diseases is that they are largely preventable and oftentimes curable, or at least can be effectively managed through appropriate medical treatment so as to avoid functional limitations. However, access to quality health care facilities and services is severely lacking in almost all regions of sub-Saharan Africa [2], where NTDs are prevalent. As a response to a shortage of financial and human resources needed to fully institute Western-style primary care structures, many countries have relied on collaborations with partner countries or nongovernmental organizations (NGOs) to carry out disease prevention programs, which employ nurses and community health care workers (CHWs) to carry out the projects’ aims. This paper will review available literature on the impact that nurses and community health care workers have had on combating NTDs and propose ways in which governmental institutions and NGOs can more effectively utilize nurses and CHW in prevention and response to NTDs

## Methods

A review of the literature using the Preferred Reporting Items for Systematic Reviews and Meta-Analyses (S1 PRISMA) was conducted to assess the role nurses and community health workers play in the primary, secondary, and tertiary prevention of NTDs in SSA. The authors collaborated with a medical research librarian to design a rigorous search protocol to identify all peer-reviewed publications on this topic. Literature searches were performed as recently as May 2016 in PubMed and Cumulative Index to Nursing and Allied Health Literature (CINAHL) databases, with no restrictions placed on language. A list of NTDs endemic to at least one SSA country was compiled from the World Health Organization’s (WHO) list of NTD programs [3] and Hotez and Kamath’s [1] ranking of NTDs in SSA by prevalence and distribution. Each disease’s name (Box 1) along with the Medical Subject Heading (MeSH) term “Neglected Diseases” was added to the search criteria. All countries in the sub-Saharan Africa region (Box 2) along with the MeSH terms “Africa, Western”; “Africa, Eastern”; and “Africa, Southern” were also included. Lastly, a variety of descriptive terms for nurses and community health workers (Box 3) were added to the search strategy. For this review, Lewin et al.’s [4] definition of a community health worker was utilized, which defines CHWs as any health worker who “perform[s] functions related to healthcare delivery, was trained in some way in the context of the intervention, but had received no formal professional or paraprofessional certification or tertiary education degree.” Articles published between January 2005 and December 2015 were reviewed in order to capture the full scope of nurses’ and CHWs’ responsibilities for NTD control within their respective countries’ health systems.

**Box 1. NTDs Searched**

- Soil-transmitted helminthiasis (STH)
- Elephantiasis (Lymphatic filariasis [LF])
- Onchocerciasis
- Schistosomiasis
- Trachoma
- Yellow fever
- African sleeping sickness (African trypanosomiasis)
- Visceral leishmaniasis
- Yaws
- Guinea worm (Dracunculiasis)
- Leprosy (*Mycobacterium leprae)*
- Buruli ulcer (*Mycobacterium ulcerans*)
- Dengue fever

**Box 2. Sub-Saharan African Countries Included in Search**

Angola, Benin, Botswana, Burkina Faso, Burundi, Cameroon, Cape Verde, Central African Republic, Chad, Congo, Cote d’Ivoire, Democratic Republic of Congo, Djibouti, Equatorial Guinea, Eritrea, Ethiopia, Gambia, Gabon, Ghana, Guinea, Guinea-Bissau, Kenya, Lesotho Liberia, Malawi, Mali, Mauritania, Mozambique, Namibia, Niger, Nigeria, Rwanda, Senegal, Sierra Leone, Somalia, South Africa, Sudan, Swaziland, Tanzania, Togo, Uganda, Zambia, and Zimbabwe

**Box 3. Community Health Worker Synonyms Searched**

- Community health worker(s)
- Community-directed drug distributor(s)
- Community health aide(s)
- Village health worker(s)
- Health extension worker

Two authors (AC and CT) separately conducted abstract and full-text searches using the described strategy. The authors then employed Dearholt and Dang’s [5] Johns Hopkins Nursing Evidence Appraisal Tool (S1 PRISMA, Appendix A) to assign Levels of Evidence and Quality (LE&Q) grades to the selected pieces of literature. Articles are assigned an evidence level ranging from I through V as well as an A, B, or C quality grade. Level I corresponds to experimental studies; level II to quasi-experimental ones; level III to nonexperimental or qualitative studies; level IV to published clinical guidelines or opinions of recognized authorities, committees, or consensus panels; and level V is assigned to nonresearch evidence such as literature reviews, quality improvement studies, case reports, or program or financial evaluations. The measurement tool poses a series of questions to the reader regarding issues involving clarity of methods, sample size sufficiency, any measurement tools’ utilized internal and external validity, the generalizability of results, synthesis of earlier literature with the present article’s findings, and study limitations. Grades of A, B, or C are assigned according to how well each article meets its Level of Evidence’s quality appraisal questions. Each article was independently appraised by the two authors and then reviewed together for concordance. Any differences between levels or grades were then discussed together. Articles with resulting lower levels of evidence or quality were not excluded from this review but, rather, results from such publications were assessed more critically if contrary to the findings of higher-scoring articles. After reviewing the literature, findings were organized using the public health model of primary, secondary, and tertiary prevention measures [6].

## Results

**Fig 1:**
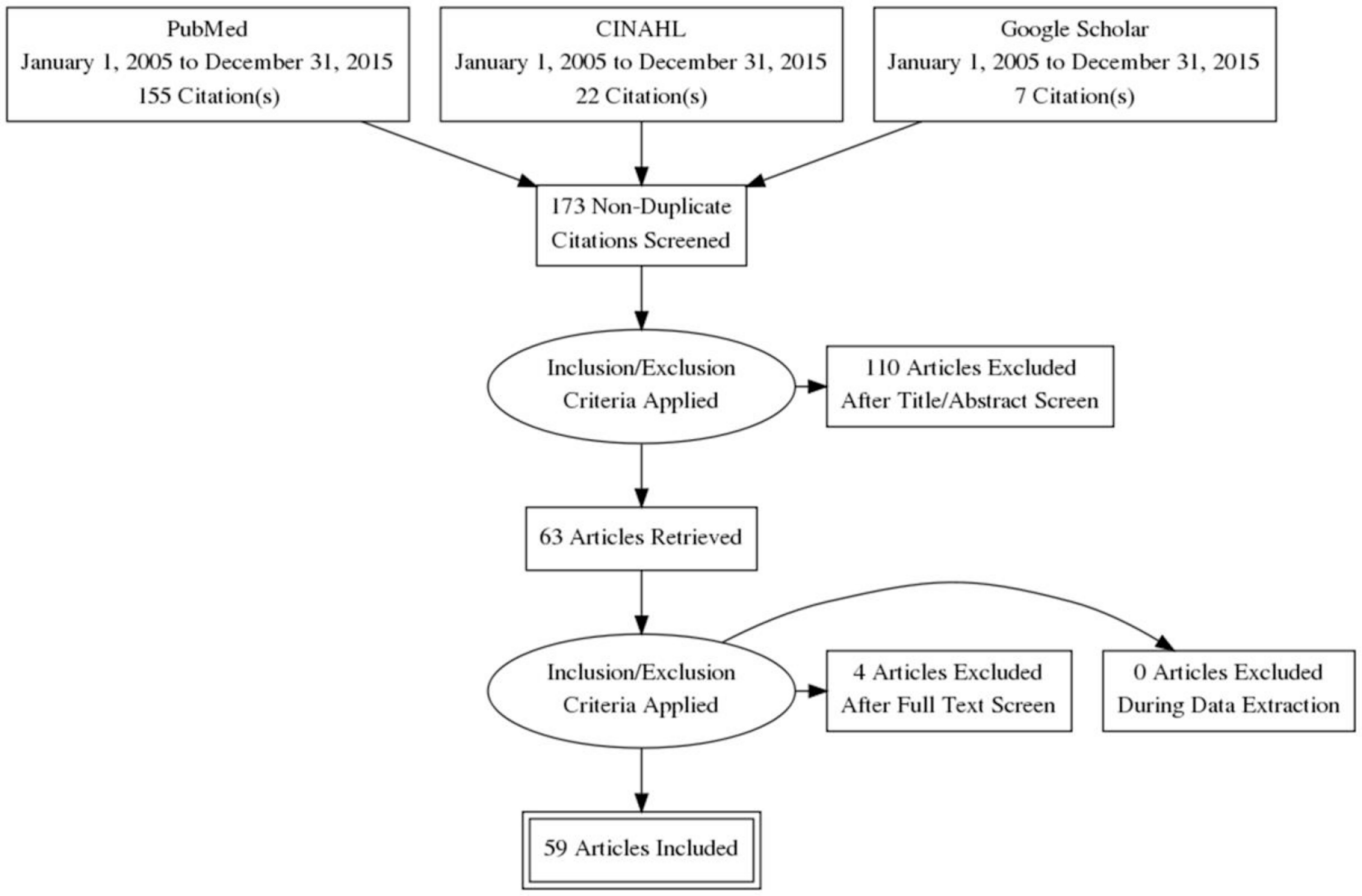
Flowchart of included and excluded literature.

A total of 155 articles were identified in PubMed and 22 in CINAHL using the above described search protocol. All entries were reviewed for relevance by reading article abstracts and, if necessary, the article body. Of the 155 entries in PubMed, 52 were identified as being relevant to the subject of nurses’ and CHWs’ roles in combating NTDs. Fifty-one of the articles identified were written in English and one article in French. Of the 22 identified in CINAHL, 6 had relevance to the subject; however, all 6 were duplicates of those identified while searching PubMed. An additional 7 publications were also identified outside the search protocol and added to the list of reviewed articles. This brought the total number of articles meeting the review inclusion criteria to 59 (Fig 1). Included articles’ title, author(s), publication year, study country or region, disease(s) studies, study structure, and LE&Q are provided in Table 1.

**Table 1:** Literature summary and Level of Evidence and Quality (LE&Q) appraisal.

**Title**	**Authors**	**Year**	**Countries or regions**	**Disease(s) studied**	**Study Structure**	**LE&Q**
Sociocultural aspects of mass delivery of praziquantel in schistosomiasis control: The Abeokuta experience	Adeneye et. al	2007	Ogun State, Southwest Nigerian	Schistosomiasis	Qualitative, semistructured interviews with male and female adolescents, children, adults, community leaders, and mass drug distributors	III, B
Assessing resources for implementing a community directed intervention (CDI) strategy in delivering multiple health interventions in urban poor communities in Southwestern Nigeria: a qualitative study	Ajayi, Jegede, Falade, and Sommerfeld	2013	Ibadan, Nigeria	Primary care interventions able to be delivered by CDI strategy	Qualitative, cross-sectional study employing focus groups and key informant interviews	III, A
Performance of predictors: Evaluating sustainability in community-directed projects of the African programme for onchocerciasis control	Amazigo et al.	2007	10 countries: Cameroon, Congo (Brazzaville), Congo (Kinshasa), Ethiopia, Malawi, Nigeria, Sudan, Tanzania, and Uganda	Onchocerciasis	Qualitative and quantitative evaluation of community-directed treatment with ivermectin (CDTI) projects using multistage random sampling. Process and result indicators were used to calculate a sustainability score.	III, A
The Impact of Nurses on Neglected Tropical Disease Management	Blood-Siegfried et al.	2014	Unspecified; underdeveloped countries	Variety of NTDs	Literature review	V, A
Capacity of community-based organizations to disseminate sleeping sickness information	Bukachi et al.	2005	Busia district, Western Kenya	Human-African Trypanosomiasis	Qualitative, purposively selected interview process administered to community-based organizations (CBOs) in a tsetse and trypanosomiasis endemic area	III, C
Interactions between Global Health Initiatives and Country Health Systems: The Case of a Neglected Tropical Diseases Control Program in Mali	Cavalli et al.	2010	Mali	Lymphatic filariasis (LF), onchocerciasis, schistosomiasis, soil-transmitted helminthiasis (STH), and trachoma	Exploratory qualitative	III, B
Monitoring of mass distribution interventions for Trachoma in Plateau State, Nigeria	Cromwell et al.	2013	Plateau State, Nigeria	Trachoma	Nonexperimental, cross-sectional survey	III, A
Factors associated with coverage of praziquantel for schistosomiasis control in the community-directed intervention (CDI) approach in Mali (West Africa)	Dabo et al.	2013	Diema, Mali	Schistosomiasis	Nonexperimental, descriptive, focused group discussions	III, A
Factors affecting the attrition of community-directed distributors of ivermectin in an onchocerciasis-control program in the Imo and Abia States of south-eastern Nigeria	Emukah et al.	2008	Nigeria	Onchocerciasis	Nonexperimental, structured interviews with community members and community-directed drug distributors (CDDs)	III, A
Task shifting for eye care in Eastern Africa: General nurses as trichiasis surgeons in Kenya, Malawi, and Tanzania	Gichangi et al.	2015	Kena, Malawi, and Tanzania	Trachoma	Nonexperimental, retrospective cohort study	III, B
Access to water source, latrine facilities and other risk factors of active Trachoma in Ankober, Ethiopia	Golovaty et al.	2009	Ethiopia	Trachoma	Cross-sectional study	III, A
Assessment of a novel approach to identify trichiasis cases using Community Treatment Assistants in Tanzania	Greene et al.	2015	Tanzania	Trachoma	Randomized control trial	I, A
Nurses engaged in the fight against leprosy	Guyon et al.	2015	Mozambique	Leprosy	Non-peer-reviewed article	V, C
Prevalence of active Trachoma two years after control activities	Hagan et al.	2009	Ghana	Trachoma	Nonexperimental, cross-sectional study employing clustered random sampling	III, C
Re-assessing community-directed treatment: Evidence from Mazabuka district, Zambia	Halwindi et al.	2015	Zambia	Helminthiasis	Nonexperimental, two cross-sectional survey periods	III, B
Socio-demographic factors associated with treatment against soil-transmitted helminth infections in children aged 12–59 months using the health facility approach alone or combined with a community-directed approach in a rural area of Zambia	Halwindi et al.	2013	Zambia	Helminthiasis	Randomized control trial, with the control receiving standard health facility (HF) services and the control receiving HF and community-directed treatment (HF+CDT).	I, B
Maintaining effective mass drug administration for lymphatic filariasis through in-process monitoring in Sierra Leone	Hodges et al.	2012	Sierra Leone	Lymphatic filiariasis	Nonexperimental comparative	III, B
Neglected tropical disease control in post-war Sierra Leone using the Onchocerciasis Control Programme as a platform	Hodges et al.	2011	Sierra Leone	Onchocerciasis, Lymphatic filiariasis, and STH	Nonresearch, organizational experience	V, B
Gender and performance of community treatment assistants in Tanzania	Jenson et al.	2014	Tanzania	Trachoma	Nonexperimental comparative	III, A
Progress towards countrywide control of schistosomiasis and soil-transmitted helminthiasis in Uganda	Kabatereine et al.	2005	Uganda	Schistosomiasis and STH	Nonresearch, program evaluation	V, B
Community-directed interventions strategy enhances efficient and effective integration of health care delivery and development activities in rural disadvantaged communities of Uganda	Katabarwa et al.	2005	Uganda	Onchocerciasis	Nonexperimental, comparative research; cross-sectional study	III, A
Monitoring ivermectin distributors involved in integrated health care services through community-directed interventions—a comparison of Cameroon and Uganda experiences over a period of three years (2004–2006)	Katabarwa et al.	2010	Uganda and Cameroon	Onchocerciasis	Nonexperimental descriptive, cross-sectional study	III, B
Traditional kinship system enhanced classic community-directed treatment with iverectin (CDTI) for onchocerciasis control in Uganda	Katabarwa et al.	2010	Uganda	Onchocerciasis	Quasi-experimental	II, B
The role of community-based surveillance in health outcomes measurement	Kyei-Faried et al.	2006	Ghana	Buruli ulcer (among a number of other infectious diseases) along with births, deaths, maternal mortality, and infant mortality	Nonexperimental descriptive	III, C
Can mobile phones help control neglected tropical diseases? Experiences from Tanzania	Madon et al.	2014	Tanzania	Variety	Qualitative case report	V, A
Effectiveness of different approaches to mass delivery of praziquantel among school-aged children in rural communities in Nigeria	Mafe et al.	2005	Nigeria	Schistosomiasis	Randomized control trial	I, B
Primary health care in rural Malawi—a qualitative assessment exploring the relevance of the community-directed interventions approach	Makaula et al.	2012	Malawi	Schistosomiasis	Qualitative; key informant interviews and group discussions	III, A
Onchocerciasis control in the Democratic Republic of Congo (DRC): Challenges in a post-war environment	Makenga Bof et al.	2015	Democratic Republic of Congo	Onchocerciasis	Nonexperimental comparative	III, A
The sharp end: Experiences from the Tanzanian programme for the elimination of lymphatic filariasis: notes from the end of the road	Malecela	2009	Tanzania	Lymphatic filiariasis	Nonresearch case report	V, B
Community perceptions on the community-directed treatments and school-based aproaches for the control of schistosomiassis and soil-transmitted helminthiasis among school-age children in Lushoto District, Tanzania	Massa et al.	2009	Tanzania	Schistosomiasis and STH	Qualitative; key informant interviews and group discussions	III, B
It is possible: Availability of lymphedema case management in each health facility in Togo. Program description, evaluation, and lessons learned.	Mathieu et al.	2013	Togo	Lymphatic filiariasis	Nonresearch, program evaluation	V, B
A community-based Trachoma Survey: Prevalence and risk factors in the Tigray Region of Northern Ethiopia	Mesfin et al.	2006	Ethiopia	Trachoma	Nonexperimental descriptive, cross sectional community-based survey using multistage cluster random sampling	III, A
Soil transmitted helminths and scabies in Zanzibar, Tanzania following mass drug administration for lymphatic filariasis—a rapid assessment methodology to assess impact	Mohammed et al.	2012	Tanzania	STH	Nonexperimental comparative, health record analysis from 50 randomly sampled primary health care units	III, A
Community directed approach beyond ivermectin in Tanzania: A promising mechanism for the delivery of complex health interventions	Mutalemwa et al.	2009	Tanzania	Onchocerciasis	Qualitative; key informant interviews and group discussions	III, B
Integrated community-directed intervention for schistosomiasis and soil transmitted helminths in western Kenya–a pilot study	Mwinzi et al.	2012	Kenya	Schistosomiasis and STH	Quasi-experimental, longitudinal	II, B
Increasing coverage in mass drug administration for Lymphatic Filariasis elimination in an urban setting: A study of Malindi Town, Kenya	Njomo et al.	2014	Kenya	Lymphatic filiariasis	Quasi-experimental	II, C
Pilot training of community mobilizers as health educators to prevent Onchocerciasis in Bugai, Kaduna State, Nigeria	Okanlawon and Osanyintolu	2012	Nigeria	Onchocerciasis	Quasi-experimental	II, C
Where would I be without ivermectin? Capturing the benefits of community-directed treatment with ivermectin in Africa	Okeibunor et al.	2011	Cameroon, DRC, Nigeria, and Uganda	Onchocerciasis	Qualitative, cross-sectional study	III, A
Community health workers' experience and perspectives on Mass Drug Administration for Schistosomiasis in Western Kenya: The SCORE Project	Omedo et al.	2012	Kenya	Schistosomiasis	Qualitative, cross-sectional	III, A
The effect of a health communication campaign on compliance with mass drug administration for Schistosomiasis control in western Kenya: The SCORE Project	Omedo et al.	2014	Kenya	Schistosomiasis	Qualitative, convenience sampling	III, A
Lymphoedema management in Africa	Penzer	2005	unspecified; sub-Saharan Africa	Lymphatic filiariasis	Clinical practice guidelines	V, C
The outcome of Trachomatous Trichiasis surgery in Ethiopia: Risk factors for recurrence	Rajak et al.	2013	Ethiopia	Trachoma	Nonexperimental, longitudinal prospective observational study	III, A
Epidemiological and entomological evaluations after six years or more of mass drug administration for Lymphatic Filariasis elimination in Nigeria	Richards et al.	2011	Nigeria	Lymphatic filiariasis	Nonexperimental, observational prospective study	III, A
Developing a community-led SMS reporting tool for the rapid assessment of lymphatic filariasis morbidity burden: case studies from Malawi and Ghana	Stanton et al.	2015	Malawi and Ghana	Lymphatic filiariasis	Quasi-experimental,stratified random sample	II, A
Community-directed interventions for priority health problems in Africa: results of a multicountry study	The CDI Study Group	2010	Uganda, Nigeria, and Cameroon	Onchocerciasis, TB, vitamin A deficiency, malaria	Randomized control trial	I, A
Uptake of mass drug administration programme for Schistosomiasis control in Koome Islands, Central Uganda	Tuhebwe et al.	2015	Uganda	Schistosomiasis	Nonexperimental, mixed methods cross-sectional study	III, B
The impact of community health workers (CHWs) on Buruli ulcer in sub-Saharan Africa: a systematic review	Vouking et al.	2013	Cameroon, Benin, Cote D'Ivoire, Ghana, and DRC	Buruli ulcer	Systematic review	III, C
The contribution of community health workers to the control of Buruli ulcer in the Ngoantet area, Cameroon	Vouking et al.	2013b	Cameroon	Buruli ulcer	Nonexperimental descriptive, cross sectional	III, B
Contribution and performance of female Community-Directed Distributors in the treatment of onchocerciasis with Ivermectin in Sub-Saharan Africa: a systematic review	Vouking, Tamo, and Tadenfok	2015	Tanzania, Nigeria, Cameroon, and Uganda	Onchocerciasis	Systematic review	III, C
Community-directed treatment of lymphatic filariasis in Kenya and its role in the national programmes for elimination of lymphatic filariasis	Wamae et al.	2006	Kenya	Lymphatic filiariasis	Randomized control trial	I, B
Buruli Ulcer in West Africa: Strategies for early detection and treatment in the antibiotic era	Webb et al.	2009	Cameroon, Benin, Cote D'Ivoire, and Ghana	Buruli ulcer	Literature review	V, B
Factors affecting community participation in the CDTI program in Morogoro, Tanzania	York et al.	2014	Tanzania	Onchocerciasis	Nonexperimental, surveys and focus group discussions	III, B
*Selected Publications*						
**Title**	**Authors**	**Year**	**Countries**	**Disease(s) studied**	**Study Structure**	**LE&Q**
Short Report: Buruli Ulcer control in a highly endemic district in Ghana: Role of community-based surveillance volunteers	Abass et al.	2014	Ghana	Buruli ulcer	Case report, retrospective analysis of clinical cases	V, A
Community-driven interventions can revolutionise control of neglected tropical diseases	Amazigo et al.	2012	nonspecific	Onchocerciasis, LF, STH	Literature review	V, A
Contribution of the Community Health Volunteers in the control of Buruli Ulcer in Benin	Barogui et al.	2014	Benin	Buruli ulcer	Nonexperimental, retrospective observational study	III, A
The potential for community-directed interventions: Reaching underserved populations in Africa	Brieger et al.	2015	n/a	multiple	Literature review	III, A
Which intervention design factors influence performance of community health workers in low- and middle-income countries? A systematic review	Kok et al.	2014	unspecified	multiple	Systematic review with meta-synthesis	III, A
Prospects of using community directed intervention strategy in delivering health services among Fulani Nomads in Enugu State, Nigeria	Okeibunor et al.	2013	Nigeria	n/a	Qualitative, in-depth, and key informant interviews	III, B
A model for evaluating the sustainability of community-directed treatment with ivermectin in the African Program for Onchocerciasis Control	Okeibunor et al.	2012	multiple	Onchocerciasis	Nonexperimental, cross-sectional study	III, A

**Fig 2:**
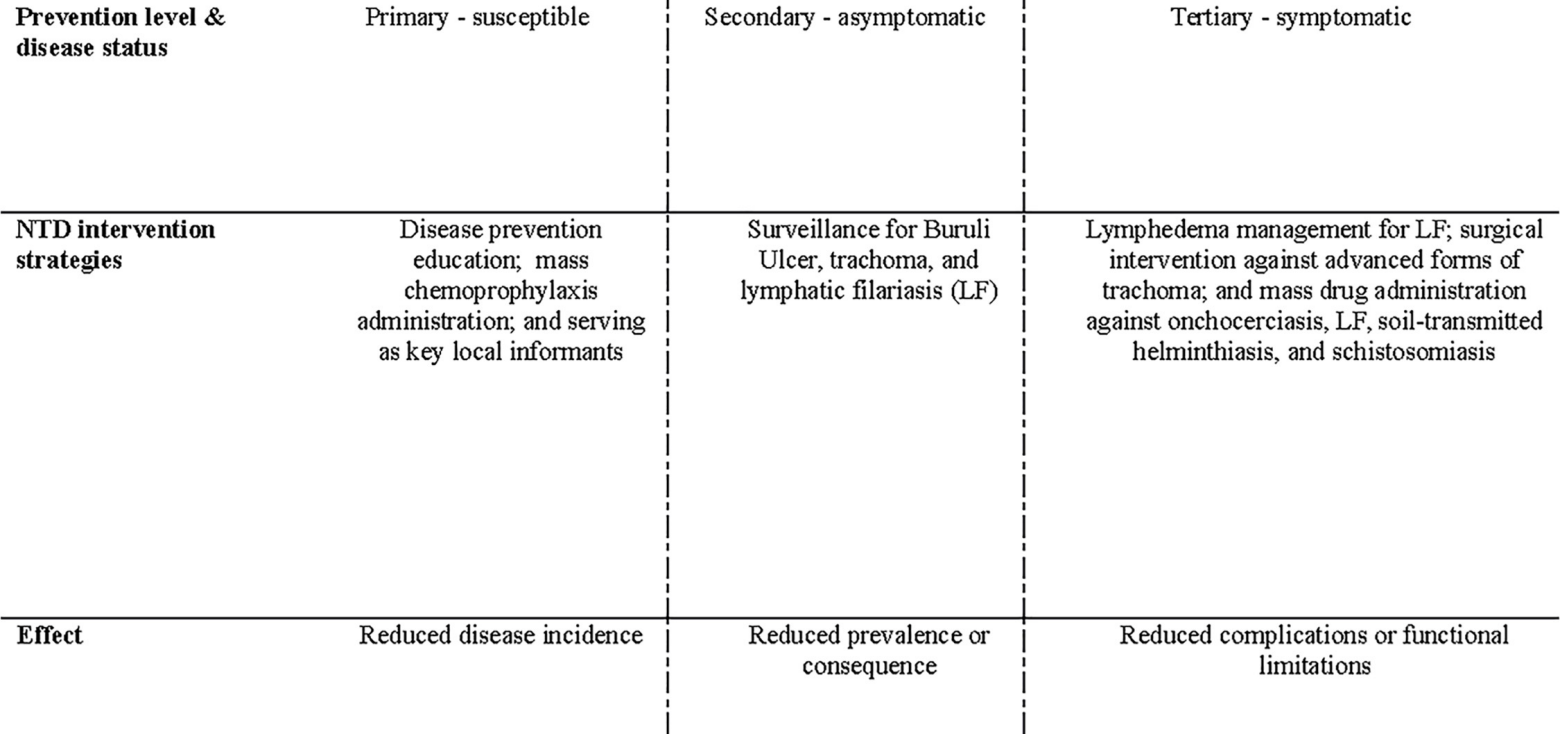
Nurses’ and CHWs’ primary, secondary, and tertiary interventions in NTD control [6].

Nurses in many SSA countries form an integral component of the professional public health and patient care workforce and engage in all parts of the NTD control spectrum. Furthermore, in many communities, nurses are the only primary care providers and serve also as trainers and managers of CHWs [7]. Success in the context of NTD control requires innovative thinking into health care delivery in order to better utilize these valuable human resources outside of health facilities [2]. The creation of corps of community health workers has been the result of such thinking, serving alongside nurses on the frontlines of health care provision in SSA and forming a bridge between health care institutions and the populations they serve. Indeed, nurses’ and CHWs’ work spans all three phases of public health intervention: primary, secondary, and tertiary prevention (Fig 2).

## Primary Prevention Activities

Trachoma, an eye infection caused by the bacterium *Chlamydia trachomatis*, is the leading cause of infectious blindness worldwide [8,9]. Since young children suffer a disproportionate burden of earlier forms of trachoma, nurses provide important advice to parents on keeping their children’s faces clean of ocular and nasal discharge, which are known risk factors for the spread of the infection [10]. Education on the etiology and seriousness of soil-transmitted helminth infections proved equally valuable in a study conducted in Zambia, in which a statistically higher proportion of children were treated with appropriate chemoprophylaxis by carers who self-reported perceiving such infections as having serious health effects than those carers who did not [11]. The authors noted that CHW work on educating client populations on the causes and effects of soil-transmitted helminth infections could improve involvement later in mass drug administration against the disease.

In a schistosomiasis control program in western Kenya, program implementers used direct feedback from CHWs to gauge the success of a public awareness campaign on the importance of participating in a mass drug administration program (MDA). In fact, the impetus for performing the communications campaign arose after experiences reported by CHWs during the first MDA indicated that certain groups were anxious about taking medications whose side effects and purpose were poorly understood [12]. In response, health education campaigns were initiated prior to the second round of MDA in order to address local concerns and anxieties. CHWs brought target audience perspectives to managers before and after the campaign, creating a unique opportunity for community-based participatory research [13]. A similar study into factors associated with participation in schistosomiasis control programs in Uganda also observed that participant involvement was more likely if the respondent was knowledgeable about schistosomiasis transmission and prevention (adjusted odds ratio [AOR] 1.85, 95% CI 1.22–2.81) and reported to have received health education from the health personnel (AOR 5.95, 95% CI 3.67–9.65) [14].

Community health workers’ knowledge of a community’s languages, customs, and context means that they are able to deliver health messages to groups in a culturally appropriate manner easily understood by them. In Kenya, community-based organizations (CBOs), such as women’s groups serving as CHWs, were found to be an effective means of disseminating information on human African trypanosomiasis (also known as African sleeping sickness) and perhaps even superior to previous top-down information dissemination programs because local groups would place a greater emphasis on community involvement, thereby improving control and surveillance program sustainability [15]. Okanlawon and Osanyintolu’s [16] work in Nigeria with CHWs trained as onchocerciasis community educators showed similar results. Following training activities, CHWs demonstrated an ability to disseminate knowledge on the disease’s prevention and treatment. With appropriate training techniques, the researchers wrote that CHWs could serve as key community mobilizers, giving fellow community members the information necessary to become better custodians of their own health.

CHWs’ value as local informants and educators was augmented when a multi-NTD control program in Tanzania began supplying CHWs with mobile phones to facilitate data capture and increase the speed at which managers were able to receive it. Managers reported that this increased data availability from CHWs in the field improved planning and decision-making capacity with problems such as quantifying the demand for drugs at each village-level MDA program. CHWs found that the phones enabled a more direct communications channel to senior health staff concerning aspects of program implementation [17].

Mass chemoprophylaxis administration is another means of primary prevention undertaken by nurses and CHWs. Lymphatic filariasis’ (elephantiasis) prevalence has through, in part, mass drug administration been effectively curbed in many previously hyperendemic regions of SSA. The Tanzanian National Lymphatic Filariasis Elimination Programme was launched in 2000 and is, in terms of geographical coverage, among one of the country’s largest disease control programs to date [18]. Programs such as Kenya’s have appeared in countries throughout SSA but rely on high drug coverage rates of the population for seven to ten years to effectively interrupt future transmission [19,20]. A description of research and methods of efficient MDA follows in “tertiary prevention.”

A lack of education of community members and CHWs was a major theme of the literature, and the importance of community education done by community nurses and CHWs is difficult to overemphasize. Educating community members empowers communities and serves to increase participation in the desired health activity. As York et al. [21] concluded when analyzing onchocerciasis control programs in Tanzania, programs’ success and sustainability is largely predicated on community sensitization.

## Secondary Prevention Activities

In many instances, nurses are tasked with identifying and diagnosing disease cases. Specially trained ophthalmic nurses using WHO’s simplified clinical grading scale are very often the first health care providers to identify and diagnose cases of trachoma [9]. Mesfin et al.’s study [9] conducted in Ethiopia of 3,900 participants used nurses to determine the prevalence of trachoma in a known endemic region. The study concluded that overall prevalence of all stages of trachoma in the study regions was higher than previously thought, and those severe forms of the disease were greater than three times the 1% cutoff for public health significance. In another study conducted in Ghana of children ages one to ten, nurses determined prevalence of active trachoma before and after a two-year mass antibiotic drug distribution intervention. Results showed an overall reduction in the five study districts ranging between 41% and 79% [10]. A randomized control trial conducted in 36 Tanzanian communities employed CHWs as trachomatous trichiasis screeners in the intervention arm of the study following a half-day training on screening techniques [22]. Results indicated that while CHWs were able to correctly rule out negative cases of the disease at a rate comparable with the control arm of the study (specificity = 96.3% versus 99.7%), they were unable to achieve as high a level of correlation with more experienced screeners on suspected positive cases as the control (positive predictive value = 15.8% versus 28.1%). In the case of trachoma, the literature seems to show that nurses are better equipped clinically to screen for the disease than CHWs.

Community health workers, however, have shown to be adept in detecting cases of lymphedema and hydrocele resulting from lymphatic filariasis. When compared against independent physician verification, CHWs in Ghana and Mozambique typically had positive predictive values greater than 90% [23].

Community health worker’s efforts in identifying cases of Buruli ulcer offer an important example on how nonprofessional health workers can be trained to detect suspected cases of an NTD, thereby increasing a public health system’s overall case detection power. Buruli ulcer is caused by the bacteria *Mycobacterium ulcerans* and is the third most common mycobacterial disease in humans [1,24]. No prophylaxis therapy exists, but the current recommended treatment of an eight-week course with streptomycin and rifampicin is considered highly effective in cases of early detection [25]. However, most ulcers are still only identified when at their more advanced stages, requiring surgical intervention along with antibiotic therapy [24]. Early detection has been identified as being vital to reducing patients’ need for surgery and hospitalization and minimizing the risk of osteomyelitis and future functional disability [24–26].

Kyei-Fareid et al. [27] suggested that a program to employ CHWs as a form of community-based disease surveillance for Buruli ulcer would help to widen the gate to health care services entry and complement institutional health rates data. They noted that this improved data would serve to show “the body of the hippopotamus” and not simply its ear alone. Webb et al. [24] also later suggested initiating community-based surveillance and education programs in endemic regions to improve the likelihood of detecting cases early on. Following a review of Buruli ulcer morbidity characteristics, the authors proposed a model in which volunteers chosen at the village level were trained as CHWs and tasked with the responsibility of active case-finding and education in several villages within a geographic zone. CHWs would make regular visits to households within their assigned zones and report to their district supervisors, often a nurse at a central district health center experienced in the clinical diagnosis of Buruli ulcer, who would confirm suspected cases. Programs similar to those proposed have been initiated in countries such as Benin and Ghana. A study conducted in Cameroon and then cited in a systematic literature review reported that 95% of participant CHWs identified and referred suspected cases of Buruli ulcer, of which 91% were confirmed cases [28,29]. In another report from Ivory Coast, results indicated that around 65% of the cases referred by CHWs were confirmed by the medical staff [28]. A retrospective study conducted in Benin of 1,965 confirmed cases of Buruli ulcer found that a quarter of all cases had been referred to health centers by trained CHWs and that these CHWs referred patients more frequently at an earlier stage of disease than the average [26]. In a similar retrospective study conducted at a clinic in Ghana during a five-year period, 45% of the 375 confirmed Buruli ulcer cases had been referred to the study clinic by CHWs, making them the single most important source of referrals. Additionally, the CHWs were more likely than other referral groups to report the disease while still in its earliest stages [25].

These same principles could prove equally useful for combating leprosy, the world’s second most common mycobacterium infection. Guyon et al. [30] offered qualitative evidence that indicated difficulty exists in addressing the disease because only those in the upper ranks of the public health service have knowledge in identifying the disease. As a result, many individuals turn first to traditional healers before the formal health sector for care, wasting valuable time, worsening their possible outcomes, and increasing the chances of functional impairment and deformation. However, in some cases, these traditional healers are also leprosy surveillance volunteers. This use of respected community members, such as traditional healers or CHWs, could be an effective means of improving surveillance.

## Tertiary Prevention Activities

Skilled nursing care is essential component to management of a number of NTDs at both the individual and population level. In regions where trachoma is a public health concern, WHO recommends the implementation of the SAFE Strategy (Surgery, Antibiotics therapy, Facial cleanliness, and Environmental change). For early-stage cases, nurses provide patients with either single-dose oral azithromycin or ophthalmic tetracycline [10]. In advanced forms of the disease, these same nurses may also conduct bi-lamellar tarsal rotation surgeries to reverse the in-turned lids and eyelashes distinctive of trachoma that cause severe scarring and opacity of the conjunctiva and eventual blindness [31,32]. Countries that offer nurses the scope of practice necessary to perform these types of surgeries should take care, however, to ensure that staff receive frequent and appropriate training. Rajak et al. [32] found that nearly a quarter (24.7%) of those having received lamellar tarsal rotation suffered recurrence. Researchers note though that recurrence rates tend to be higher in those nurse-surgeons who performed the procedure only infrequently, an argument for frequent in-service training and practice [33].

Education on managing the chronic lymphedema associated with lymphatic filariasis is an important component of care and includes nurses ensuring patients are knowledgeable on how to properly care for affected extremities as well as able to identify further coinfection risks [34]. In Togo, a national lymphatic filariasis management program was designed around dispensary-level nurses whose responsibility it was to educate patients on lymphedema self-care and to train CHWs in this same lymphedema management so that they could later return to patients’ homes to assess their progress and offer moral support and motivation [35].

While the management of both acute and chronic NTD-associated conditions is an important activity undertaken by CHWs, seemingly the single most intensely studied aspect of CHWs’ role in tertiary prevention of NTDs is as mass drug distributors, and the NTD most commonly associated with such an intervention is onchocerciasis. Onchocerciasis (river blindness) is a parasitic disease caused by a filarial worm and spread by blackflies, which breed in fast-flowing streams and rivers. As its name suggests, the disease can cause eventual blindness, but it is also the source of severe skin disease and near-debilitating itching [36]. Onchocerciasis creates a constellation of problems that amount to a grave development obstacle for SSA, where 75 million people are at risk and 99% of those infected live [1,16,37]. In 1987, Merck & Co., Inc. pledged to supply ivermectin, the medication used to treat and ultimately halt transmission of onchocerciasis, free of charge to all those at risk until the disease had been eliminated. At the time, though, an effective means of distributing the medication to remote areas of SSA, many far from urban health centers, had not been established. In order for onchocerciasis to be eliminated from a region, it was estimated that drug distribution to 65% of the population must be maintained for 15 to 18 years [37,38]. Through the early 1990s, WHO undertook a series of studies to identify a means of distributing the medication to populations at risk, and in 1997, WHO’s African Programme for Onchocerciasis Control (APOC) formally adopted the community-directed treatment with ivermectin (CDTI) strategy [39]. In the CDTI approach, community members actively discuss the health and developmental impact of onchocerciasis based on their own experiences and information provided to them by the initiators or experts; review possible interventions and design an approach to implement them in the community; identify resources needed to carry out the interventions; and plan how, when, where, and by whom they will be implemented [40]. During this process, the community selects those who will serve as community-directed drug distributors (CDDs). While the medication is provided free of charge, it is the community’s responsibility to offer logistical, financial, and in-kind support in order to accomplish the task of drug distribution. Having local communities select CDDs from their region and expecting broad community involvement is thought to create a stronger sense of local ownership than top-down distribution strategies and has been recognized as one of the reasons for the program’s success and sustainability [37,40,41]. Today, the African-based APOC CDTI program is used in 31 NTD-endemic countries and has led to treating a reported 100 million people in 2014 [42].

The success of the CDTI strategy has been noted by other disease program implementers, who see the program’s framework as one into which their own intervention strategies could be integrated. As such, major NTD and other health-related co-implementation programs have been established in many SSA countries and are now being carried out by APOC CDDs. These programs include drug distribution interventions against soil-transmitted helminthiasis (STH), lymphatic filariasis, schistosomiasis, and trachoma; vitamin A supplementation; preventive eye care; malaria home management and distribution of insecticide impregnated bed nets; and poliomyelitis and measles immunization campaigns [43].

A study of the use of a community-directed intervention (CDI) strategy against schistosomiasis in western Mali showed that CHWs were able to attain coverage rates in excess of 75%—the theorized threshold to interrupt transmission—among school-aged children in areas with the highest CHW-to-client population ratios. Notably, coverage rates were significantly lower among the Fulani and Moorish ethnic populations, who were typically served by CHWs from the local settled populations [44]. Disparities in intervention coverage rates among these mobile pastoralists as well as urban populations is a theme found throughout much of the literature [45–49], and one that will be further explored in the Discussion section. In Nigeria, researchers have found that when comparing different schistosomiasis control programs, those offering their target communities greater decision on where and when to distribute medication had higher coverage rates [50,51].

Soil-transmitted helminthiasis (STH), a group of parasitic worm infections caused by feces-contaminated soil, is estimated to have the greatest disease burden of all neglected tropical diseases [1] but is one that can also be treated using an MDA modality. A comparison of a traditional health facility distribution scheme to distribution of antihelminthic agents through a program incorporating a CDI method calculated that the traditional method of distributing drugs from a single, fixed location attained approximately 38% coverage of the target population, while the community-directed intervention reached nearly 65% coverage [52]. Likewise, pretreatment coverage of qualifying populations with antihelminthic medications in a study of western Kenya communities averaged 17.4%. Following the implementation of a CDI distribution strategy, though, rates increased to 54.1%–96.6% [53]. A similar trend was found in Tanzania when comparing school-based distribution and CDD programs for schistosomiasis and STH medications. While both methods attained coverage of around 80% in children enrolled in school, only the CDD program attained 80% coverage in nonenrolled school children, while the school-based distribution program’s coverage dropped to around 59% [54].

A strong sense of community ownership, as demonstrated through local-level selection of drug distributors and meaningful support of CDDs’ work, maintaining high ratios of CHWs to the population served, maintaining a healthy CDD gender mix, and close proximity of CHWs’ residences to where their client population lives are common themes throughout the literature and frequently cited as hallmarks of strong CDTI programs. These themes align closely with the seven aspects of CDTI sustainability identified by Okeibunor et al. [55]: efficiency, integration, resources, health staff acceptance, effectiveness, community ownership, and simplicity. Amazigo et al. [37] noted when studying CDTI projects in ten different SSA countries that communities that planned their own distribution activities and had high CHW-to-client population ratios achieved higher sustainability scores. In a survey of 101 current and former CDDs in Nigeria, researchers found that CDD attrition was lower amongst those who were selected during community meetings than those selected by councilors from the local health facility [56]. Indeed, studies on NTD control programs from Nigeria [56], Mali [44], Uganda [57,58], Tanzania [17,59], and a number of other countries all credit strong community support and high numbers of CHWs as contributing to high levels of CHW productivity.

In economic terms, the CDI approach to drug distribution, namely against onchocerciasis, has been acknowledged as having increased food security for many SSA countries and served as a tool of poverty reduction [60]. Moreover, retrospective data from countries that have maintained CDTI programs for several years are able to achieve notable economies of scale. In the Democratic Republic of Congo, the cost of treatment is calculated to have fallen from US$1.10 in 2001 to US$0.10 in 2012, while therapeutic coverage increased from 2.7% to 74.2% during those same years [61]. However, single or even multidisease treatment programs using community-directed strategies for MDA and related interventions should not be viewed as a panacea for countries’ poorly equipped or underperforming health systems. Large programs risk diverting national time, resources, and attention away from other important public health issues. [62] A discussion of responsible program integration follows in the Discussion section.

The African Programme for Onchocerciasis Control—the organization who pioneered this strategy—however, discontinued its operations in 2015. In its place, the Program for the Elimination of Neglected Diseases in Africa (PENDA) will take on the wider mandate of eliminating all five NTDs for which pharmacotherapeutic agents exist (onchocerciasis, lymphatic filariasis, trachoma, schistosomiasis, and soil-transmitted helminthiasis) [42]. The CDI method for MDA has been shown to be an effective means to distribute drugs. The once-novel idea of involving communities in the decision-making process in order to promote local ownership has fast become a model for not only disease prevention organizations but also the larger international development community.

## Discussion

Along with descriptions of the specific roles that nurses and CHWs occupy when intervening against NTDs, the literature is replete with examples of the challenges and opportunities that countries and NTD prevention and treatment programs face when attempting to more efficiently employ limited human and financial resources and increase intervention coverage rates. Three notable themes were workforce considerations, the challenge of reaching mobile pastoralist and urban populations, and program integration and horizontal program planning (Box 4).

**Box 4. Discussion Key Points**

**Workforce considerations**

- SSA countries should ensure that their nurses’ education and scope of practice are sufficient for the NTD control measures they are asked to undertake.
- Program managers must consider the same workforce factors for CHWs as they would with formal health care personnel.

**Treating urban and mobile pastoralist populations**

- Ethnic diversity and a weaker social fabric are theorized barriers to increasing intervention coverage rates in urban populations.
- Health structures ill equipped to meet mobile pastoralist populations’ needs create a barrier to their treatment.

**Intervention integration and horizontal program planning**

- Vertical disease control programs, while potentially effective, can affect the capacity of already strained health systems to care for other conditions and populations.
- Combining multiple NTD control programs and better integrating such programs into countries’ primary care systems are strategies that could serve to create further service delivery efficiencies and improve overall capacity.

## Workforce Considerations

Nurses are a key component of most countries’ health care workforce. In sub-Saharan Africa, local-level nurses’ combination of formal biomedical training and patient care techniques and an understanding of their communities’ needs make them valuable resources and ideal program managers and trainers of CHWs involved in NTD prevention, treatment, and control services. It is critical though that nurses receive appropriate training to complete the tasks they are asked to perform in the service of their patients. A study from Ethiopia found entropion (in-turning of the eyelid) recurrence in nearly 25% of patients who received lamellar rotation surgery from ophthalmic nurses [32]. Nurses expected to perform advanced interventions such as these or other programmatic management roles should also expect appropriate training.

Despite the focus placed on them by this article, NTD prevention is only one of many competing health care initiatives that require nurses’ time, effort, coordination, and manpower [7]. As such, there remains a major need for expanding the nursing workforce in SSA. Important questions regarding cost effectiveness, nurse education, nurse retention through financial and nonfinancial motivation, and augmenting nurses’ scopes of practice to account for the important role they often play in developing SSA countries must be addressed [63].

Consideration must also be given to how CHWs are employed. Factors such as level of training, self-reported job satisfaction, gender mix, financial incentives, acceptance of CHWs by target community and partnering health care professional stakeholders, and CHW-to-population ratios are all factors associated with CHW productivity and attrition [41,44,50,56,64–67]. High attrition and low morale can quickly deplete a program’s budget and severely limit its potential impact. Program managers should seek to treat their CHW workforce as a valuable human resource that can either make or break an intervention.

## The Challenges of NTD Treatment among Urban and Mobile Pastoralist Populations

Most articles addressed NTD control in the context of rural, ethnically homogeneous populations. However, it is estimated that as of 2014, 37% of SSA’s population live in urban environments [68]. These largely unplanned, ethnically and linguistically diverse urban areas create new challenges for all health promotion programs. Previous studies have credited low urban MDA coverage rates with challenges such as population registration prior to MDA because of the presence of nonresident populations; limited accessibility of the urban dwellers to receive door-to-door treatment; the necessity to acquire specific parental consent; low awareness of ongoing MDA programs; differences in income levels; rapid expansion of urban areas and rural-to-urban mobility cycles; and, to a lesser extent, language differences [12,46,47]. Additionally, the social prestige that many programs rely on to attract and maintain volunteer CHWs might be a stronger factor in rural settings because of the existence of more stable communities with stronger social fabric than compared to more unstable urban slum communities [66].

Creating sustainable NTD control programs designed to serve mobile pastoralist populations has proven to be equally irksome. Sixty percent of the world’s 50–100 million mobile and semimobile pastoralists live in Africa and constitute a large proportion of many SSA countries’ populations. These groups are also disproportionately vulnerable to infectious diseases and experience lower coverage rates of MDA, in part, because current health structures are poorly designed to meet their needs [44,48].

Despite having been initially developed for onchocerciasis control, CDI methods could also prove to be a promising method of providing primary care interventions to hard-to-reach populations with no prior CDTI structures. The UNICEF/UNDP/World Bank/WHO Special Program of Research and Training in Tropical Diseases undertook studies to explore the feasibility of introducing new CDI among mobile pastoralists and urban poor populations. Later analysis of these studies by Brieger et al. [45] and Ajayi, Jegede, Falade, and Sommerfeld [47] indicated that CDI and other community participatory mechanisms could form the basis of viable, locally led, primary care structures. Hodges et al. [67] speculated that on a rapidly urbanizing continent, continued migration could affect the success of future MDAs unless innovative strategies were employed: going to work sites to treat employees with the collaboration of employers' medical teams or labor and employment agencies. This would mark a move away from maintaining traditional village CDIs and MDAs under the control of public health structures. Despite some literature existing on the matter, there is still a need to further investigate the challenges unique to NTD control in rapidly expanding urban communities and still poorly served mobile pastoralist groups. Further research into engaging with these hard-to-reach populations is warranted.

## Intervention Integration and Horizontal Program Planning

As is evidenced by the literature, there exist a great many NTD control programs in SSA. Creating focused initiatives can be an effective means of countering the effects of a disease on a population. However, these vertical disease-by-disease programs are often limited in scope, uneven in distribution, conflict with one another for local manpower, and tend to ignore the multitude of other health issues target communities face [62,69]. Also of concern, interest groups and NGOs will often hire local nurses to support specific interests of their funding organizations, which encourages nurses to spend more of their time on these special programs at the risk of neglecting their normal nursing duties [7]. Ultimately, these well-intentioned but somewhat myopic disease promotion programs are an inefficient means of delivering public health interventions and have even been accused of further fragmenting countries’ larger health systems, causing stress on many SSA countries’ already strained health workforces [70].

One solution to this dilemma is for SSA countries to create through their ministries of health national NTD control programs, such as Sierra Leone, Tanzania, and Malawi have all already begun building [17,67,69]. Such national control programs would serve as coordinators of a country’s major NTD control programs, putting administration at the national level while specific implementation decisions remained at the local level. NTD control programs would also facilitate information and best practices exchanges between different disease control efforts as well as serve as powerful advocates for the control and treatment of these enormous health burdens on SSA. Creating multi-NTD control administrations would likely result in CHWs being engaged in control efforts for multiple targeted NTDs. While the literature offers a mix of opinions on whether CHWs’ performance on any one program is affected when engaged in multiple disease control programs, there is evidence to suggest that integrating multiple disease control programs leads to efficiencies in population coverage for all targeted diseases. A health records analysis of 50 primary health care sites in Tanzania to assess the impact of lymphatic filariasis mass drug administration on soil-transmitted helminthiasis diagnosis rates at the health centers indicated that over the five years of records analyzed, there were reductions in diagnosed cases of STH in all age groups ranging from 89% to 97% [71]. Moreover, in regions of Liberia endemic for onchocerciasis, LF, STH, and schistosomiasis, Ministry of Health officials have found success integrating additional pharmacotherapeutic agents into existing onchocerciasis CDTI programs so as to target all four diseases simultaneously [49].

Along with the creation of national-level advocacy programs for NTD control, countries must look to further integrate the management of such diseases into their larger primary care systems. Because of a lack of health care professionals and the expense of providing universal primary health care, Malawi has taken such a similar step by integrating schistosomiasis control into its Essential Health Package (EHP), a suite of eleven basic health interventions available to all Malawians. Follow-up research determined through qualitative analysis that through the community-driven CDI approach, EHP services were being provided more effectively than compared to centralized distribution methods [69]. The CDI Study Group [70] conducted three-year-long randomized control trials in Nigeria, Cameroon, and Uganda to evaluate the efficiency and cost of integrating well-established primary care health interventions (vitamin A supplementation; distribution and retreatment of insecticide-treated bed nets; detection and referral of tuberculosis cases and short course, directly-observed treatment; and home malaria management) into existing CDTI programs. Second- and third-year coverage rates indicated that coverage rates for vitamin A supplementation, insecticide-treated nets distribution, and home malaria management were notably higher when delivered through the CDI process. Increases in coverage were especially marked for the antimalarial interventions, which were more than double the rates observed in control sites. Lastly, the median cost for delivering all five interventions through the CDI experiment sites was reported as being half that of delivering them at the control sites [70].

## Limitations

Literature concerning the role that nurses and CHWs play in preventing and treating yaws, yellow fever, dengue fever, Guinea worm, or visceral leishmaniasis was not identified. Additionally, few high-quality randomized control trials exploring alternative nurse or CHW-led surveillance or drug distribution methods were available for analysis. As such, comparing the effectiveness of different methods of engaging with hard-to-reach populations was limited to a handful of studies.

## Conclusion

The last decade of collective experience in NTD control in SSA have taught country health officials, researchers, and program managers a number of key lessons: (1) successful disease control requires deep and meaningful engagement with local communities; (2) expanding the role of nurses and CHWs will be required if SSA countries are to meet NTD treatment goals and eliminate the possibility future disease transmission; and (3) horizontal disease control programs can create complementary interactions between their different control activities as well as reduce costs through improved program efficiencies—benefits that vertical programs are not able to attain. As Makaula et al. [69] suggest, while diseases such as measles and HIV/AIDS require substantial medical training to properly manage, there exists a growing body of literature to suggest that interventions against conditions such as malaria, diarrheal diseases, malnutrition, and a whole host of NTDs can realistically be managed at the community level by well-trained and well-managed CHWs. Therefore, future NTD control programs should be thoughtfully crafted, well-integrated horizontal programs that operate parallel to and in harmony with other primary care health efforts.

**Key Learning Points**

- Frontline nurses and community health workers have contributed to the control and near eradication of a number of different NTDs and been employed in a variety of different roles all along the public health prevention continuum.
- Meaningful engagement and participation by communities in NTD prevention efforts increases programs’ likelihood of success and sustainability.
- Horizontal disease control programs have the potential to create complimentary interactions between their different control activities as well as reduce costs through improved program efficiencies—benefits that vertical programs are not able to attain.

**Top Five Papers**

1. Amazigo, U. V., Leak, S. G., Zoure, H. G., Njepuome, N., & Lusamba-Dikassa, P. S. (2012). Community-driven interventions can revolutionize control of neglected tropical diseases. Trends in Parasitology 28(6): 231–238.
2. Amazigo, U., Okeibunor, J., Matovu, V., Zouré, H., Bump, J., & Seketeli, A. (2007). Performance of predictors: Evaluating sustainability in community-directed treatment projects of the African programme for onchocerciasis control. Social Science & Medicine 64(10): 2070–2082.
3. Brieger, W. R., Sommerfeld, J. U., Amazigo, U. V., & The CDI Study Group. (2015). The potential for community-directed interventions: Reaching underserved populations in Africa. International Quarterly of Community Health Education 35(4): 295–316.
4. Katabarwa, M. N., Habomugisha, P., Richards, F. O., & Hopkins, D. (2005). Community-directed interventions strategy enhances efficient and effective integration of health care delivery and development activities in rural disadvantaged communities of Uganda. Tropical Medicine & International Health 10(4): 312–321.
5. The CDI Study Group. (2010). Community-directed interventions for priority health problems in Africa: Results of a multicountry study. Bulletin of the World Health Organization 88(7): 509–518.

## Supporting Information

S1 PRISMA PRISMA Checklist. (DOCX)
